# Impact of Undergoing Thoracolumbar Surgery on Patient Psychosocial Profiles

**DOI:** 10.1177/21925682231191693

**Published:** 2023-07-28

**Authors:** Samantha Rogers, Neil Manson, Erin Bigney, Rory McPhee, Amanda Vandewint, Eden Richardson, Dana El-Mughayyar, Edward Abraham

**Affiliations:** 1 471849 Dalhousie Medicine New Brunswick, Saint John, NB, Canada; 2 Canada East Spine Centre, Saint John, NB, Canada; 3 Saint John Orthopaedics, Saint John, NB, Canada; 4 10068 Horizon Health Network, Saint John, NB, Canada; 5 University of New Brunswick, Fredericton, NB, Canada; 6 University of New Brunswick, Saint John, NB, Canada; 7 Canadian Spine Outcomes and Research Network, Markham, ON, Canada

**Keywords:** thoracolumbar, surgery, back pain, disability, mental health

## Abstract

**Study Design:**

Prospective cohort study.

**Objective:**

Investigate the impact of thoracolumbar surgery on patients’ psychosocial profiles.

**Methods:**

A prospective cohort study of thoracolumbar surgery patients (N = 177). Measures of interest collected at baseline and 24-months after surgery were: modified Oswestry Disability Index (mODI), Numerical Rating Scores for Back Pain (NRS-B), Leg Pain (NRS-L), Pain Catastrophizing Scale (PCS), Tampa Scale of Kinesiophobia (TSK), Chronic Pain Acceptance Questionnaire-8 (CPAQ-8), Multidimensional Scale of Perceived Social Support (MSPSS), Mental Component Summary (MCS) and patient expectations for surgery impacts on mental well-being. Cohorts were separated based on attaining meaningful change defined as either 30% improvement or minimal scores in NRS-B, NRS-L and mODI. Mixed measures ANOVAs were run (α = .05).

**Results:**

Patients who showed meaningful change had significant improvements in PCS, TSK and CPAQ-8 scores but not in MSPSS scores. Patients had improvement in MCS scores over 24-months follow-up, but this change was not significantly different based on attainment of meaningful change. Overall, 75.9% of patients reported their mental well-being expectations were met. Patients who did not achieve meaningful change showed no change on any psychosocial measures with only 55.9% reporting their mental well-being expectations met.

**Conclusion:**

Thoracolumbar surgery results in significant improvement of psychosocial variables for patients who experienced meaningful change for pain and disability. Worsening of psychosocial health was not evident in patients who did not attain meaningful change.

## Introduction

Psychological factors are important to consider in surgical populations and there is a growing body of literature investigating the role of baseline psychological factors on surgical outcomes and post-surgical quality of life.^1-3^ The literature has mainly focused on psychological factors as predictors for surgical outcomes. Few studies have looked at this relationship in reverse and examined the impact of surgical outcomes on psychological health. One study reported that patients with poor outcomes following spine surgery had increased rates of depression and maladaptive beliefs^4^ but they did not evaluate specific psychometric scores. Psychological factors with evidence of impact in patients undergoing orthopaedic surgery include pain catastrophizing, kinesiophobia, social support, chronic pain acceptance and general mental health.^1,2,5,6^ A better understanding of how spine surgery could be affecting these psychological measures could help the surgeon and the patient manage surgical expectations.

Patient expectations are correlated with patients’ postsurgical satisfaction.^7^ It has not been clearly delineated whether patients undergoing spine surgery expect to see improvement in mental health, and if surgery fulfills these mental health expectations.

The objectives of the current study are to: (1) determine if a range of psychometric scores (pain catastrophizing, kinesiophobia, chronic pain acceptance, social support, and general mental health) differ between groups of patients who did and did not reach parameters for meaningful change from baseline to 24-months after surgery, and (2) determine the proportion of patients who expect to have an improvement in their mental health after surgery, and what proportion of those patients report fulfillment of that expectation.

## Methods

### Study Design and Participants

Patients (N = 177) who underwent elective thoracolumbar surgery at a single tertiary care center and were enrolled in the Canadian Spine Outcomes and Research Network (CSORN), a national registry of spine patients. Exclusion criteria were patients who underwent surgery due to traumatic fracture or tumor. All participants provided written informed consent prior to enrolment in the study. Ethical approval was provided by the Horizon Health Network Research Ethics Board (file #100841). All methods were carried out in accordance with these guidelines and regulations.

Patient demographics are reported as counts and percentages for categorical variables and means with standard deviations for continuous variables. Measures of interest included: the modified Oswestry Disability Index (mODI), the Numerical Rating Scores for Back Pain (NRS-B) and Leg Pain (NRS-L), the Pain Catastrophizing Scale (PCS), the Tampa Scale of Kinesiophobia (TSK), the Chronic Pain Acceptance Questionnaire-8 (CPAQ-8), the Multidimensional Scale of Perceived Social Support (MSPSS), the Mental Component Summary (MCS) and patient expectations for surgery impacts on mental well-being.

## Measurements

### Leg Pain and Back Pain Intensity

Leg pain intensity and back pain intensity were measured using the validated Numeric Rating Scale (NRS).^8^ This is a numeric 11-point scale that rates the patient’s subjective pain intensity on a scale from 0-10, where 0 indicates no pain and 10 indicates the worst pain they’ve ever experienced.^9^

### Disability

Disability due to leg and/or back pain was quantified using the mODI, where patients rated their difficulty with 10 daily activities. From these questions, patients receive a total score out of 100, where 0-22 is classified as minimal disability, 23-40 is moderate disability, 41-60 is severe disability, 61-80 is crippled and 81-100 is bed-bound or exaggerating.^10,11^ The mODI is a is a valid and reliable scale; internal consistency ranges from .71 to .87.^12^

### General Mental Health

The Mental Health Component Scale (MCS) is a validated and reliable (α = .76-.77) measure to assess a patient’s overall mental health and is a component of the Short Form Health Survey 12 (SF-12) which measures overall patient health.^13,14^ The MCS measures vitality, social functioning, emotional and mental health.^14^

### Pain Catastrophizing

Pain catastrophizing was quantified using the reliable PCS (α = .87) in which the patients answered 13 statements related to rumination and catastrophizing of pain.^15^ Patients received a total score out of 52 where higher scores indicate increased pain catastrophizing.

### Kinesiophobia

The TSK measures kinesiophobia on a 4-point scale ranging from strongly disagree to strongly agree.^16^ The 17 items include statements related to fear of movement. A higher score indicates a higher level of kinesiophobia. The TSK is a reliable scale for assessing kinesiophobia (α = .77).^17^

### Chronic Pain Acceptance

The Chronic Pain Acceptance Questionnaire-8 (CPAQ-8) is a valid shortened version of the original CPAQ allowing for quantitative assessment of chronic pain acceptance.^18,19^ It is an 8-item questionnaire from which participants rate their level of agreement on a scale of 0-6. A higher score indicates a higher level of chronic pain acceptance. The CPAQ-8 has a reliability of .77 to .89.^19^

### Social Support

Social support was quantified using the MSPSS which assesses support from friends and family.^20^ Patients rated their perceived level of social support using 12 statements on a 7-point scale ranging from “strongly disagree” to “strongly agree”. A higher MSPSS score indicates more social support. The MSPSS is a reliable scale for measuring perceived social support (α = .84-.92).^21^

### Patient Expectations for Mental Health Improvement

Patients were asked to rate the expected degree of change to their mental health as a result of surgery. They could respond “much better”, “better”, “somewhat better”, “no change” or “I don’t know”. Patients who responded “much better”, “better” or “somewhat better” were collapsed and labeled as expecting surgery to improve their mental health. At 24-months follow-up, patients were asked how surgery fulfilled their expectations for improved mental health. Patients could respond “yes completely”, “somewhat”, “not at all” or “I don’t know”. Patients were considered to have their expectations met at 24-months if they answered “yes completely” or “somewhat”.

## Defining Surgical Outcome

The literature has shown that a statistically significant change in post-surgical outcomes does not always translate to a significant change in the patient’s life.^22^ Within spine surgery research, it has been suggested that a 30% decrease in NRS or mODI scores is the minimal clinically important difference.^22^ Patients were organized into cohorts based on whether they demonstrated meaningful change following surgery. A meaningful change was quantified as a 30% reduction in NRS pain scores or mODI scores between baseline and 24-months follow-up or follow-up scores below the minimal pain (≤3) or disability (≤22) values. A non-meaningful change is quantified as less than a 30% reduction in NRS pain scores or mODI scores between baseline to 24-months follow-up.

## Data Analysis

A repeated measures ANOVA was used to determine how the psychometric scores changed in all patients who underwent surgery. This provided an indication of how the surgical group did, regardless of surgical outcome. A mixed measures ANOVA was used to determine if there are significant differences in psychometric scores between surgical groups (experienced meaningful change or not) and within a surgical group (at baseline and 24-months follow-up) as well as to identify any interaction that may be present within these groups. The frequency of patients who reported that their expectations were met regarding their mental health was determined for both the surgical groups. A 2 × 2 Chi square analysis was used to report differences in expectations between the 2 surgical groups.

## Results

**Figure 1.**
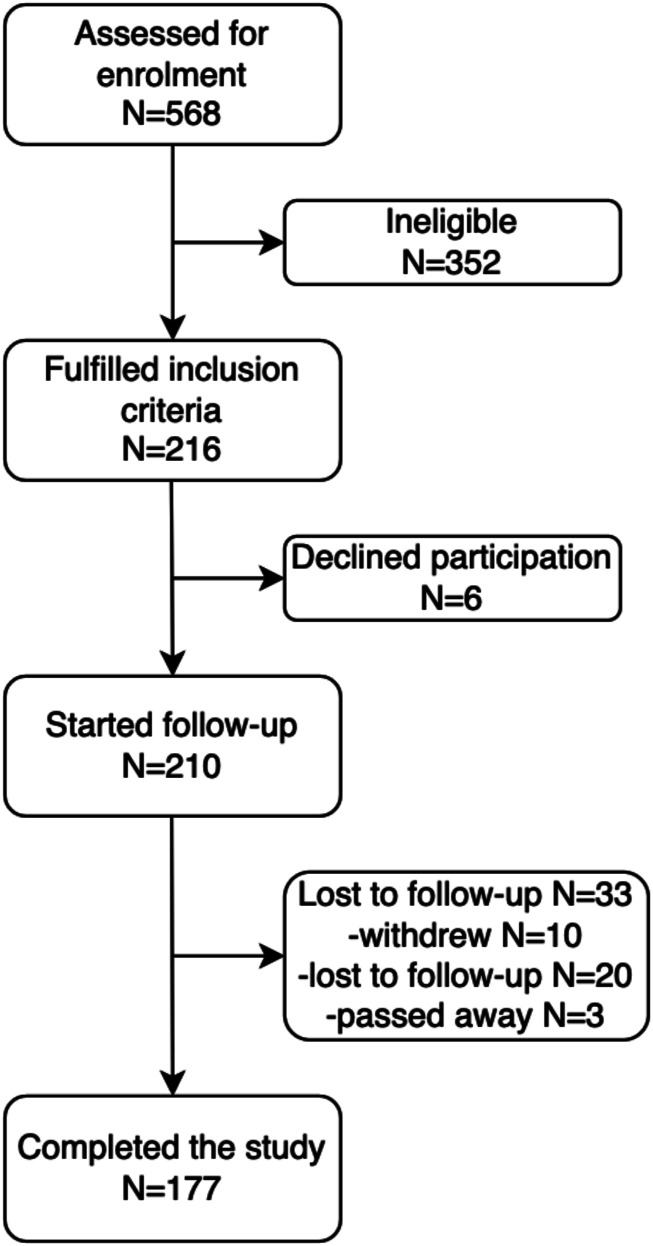
STROBE flowchart for patient enrolment and attrition. This figure shows the sample size from initial enrolment to final inclusion.

Of the 210 patients who met criteria for this study, 10 participants withdrew, 20 were lost to follow-up, and 3 passed away of unrelated causes. This is shown in Figure 1. Patients must complete all questions on the psychological scales in order to compute a total score for that scale. As such, 44 patients (24.9%) were not included in the main analysis due to missing data on core psychometric measures. There were 177 participants in the final sample utilized in the main analysis.

**Figure 2.**
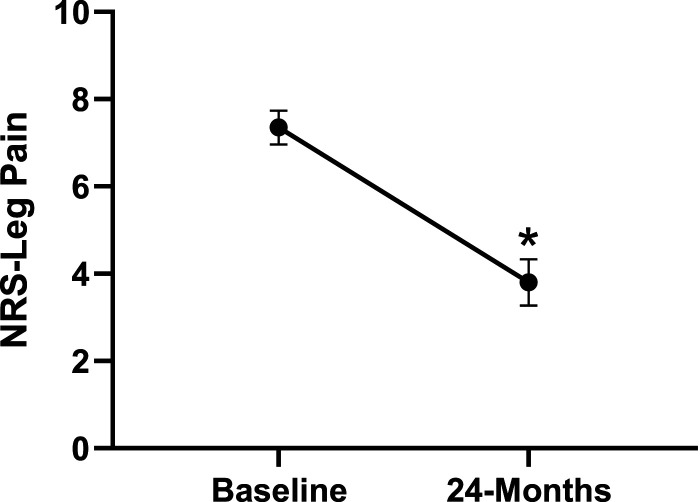
Change in leg pain from baseline to 24-months follow-up. Error bars represent confidence intervals. *Abbreviations*: NRS, Numeric Rating Scale. *P < .05.

**Figure 3.**
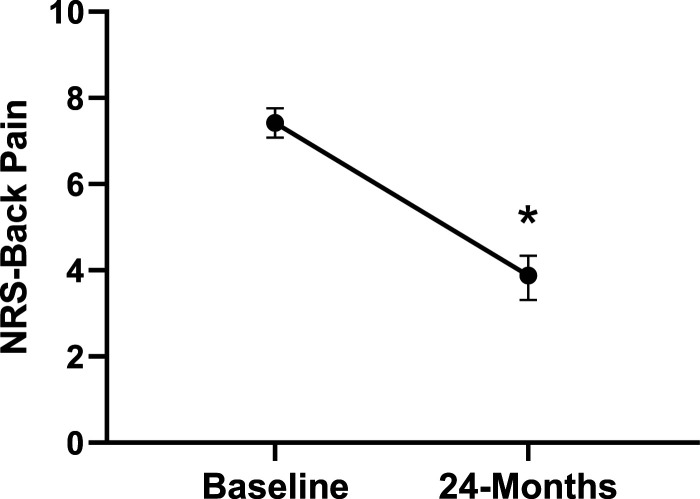
Change in back pain from baseline to 24-months follow-up. Error bars represent confidence intervals. *Abbreviations*: NRS, Numeric Rating Scale. *P < .05.

**Figure 4.**
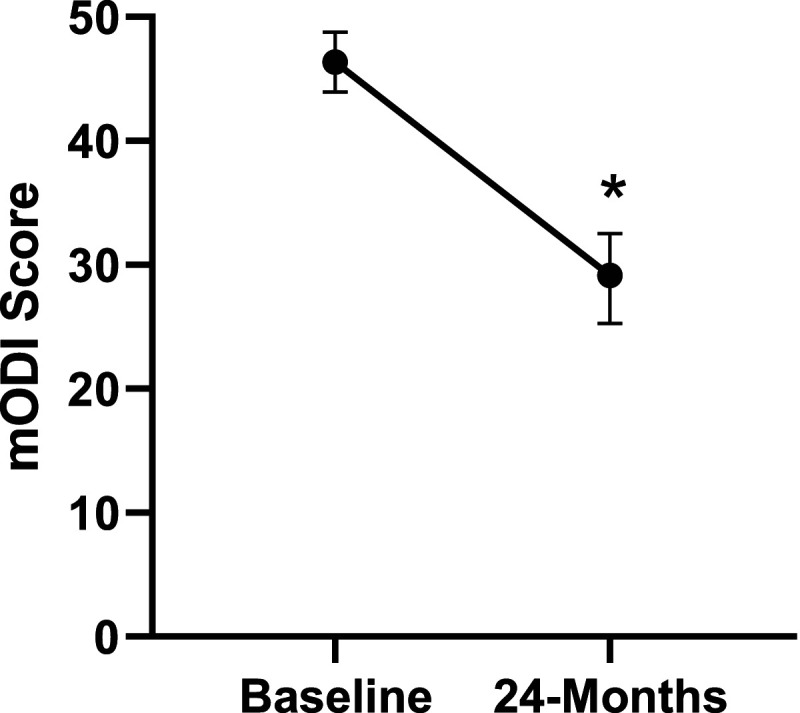
Change in disability from baseline to 24-months follow-up. Error bars represent confidence intervals. *Abbreviations*: mODI, modified Oswestry Disability Index. *P < .05.

**Figure 5.**
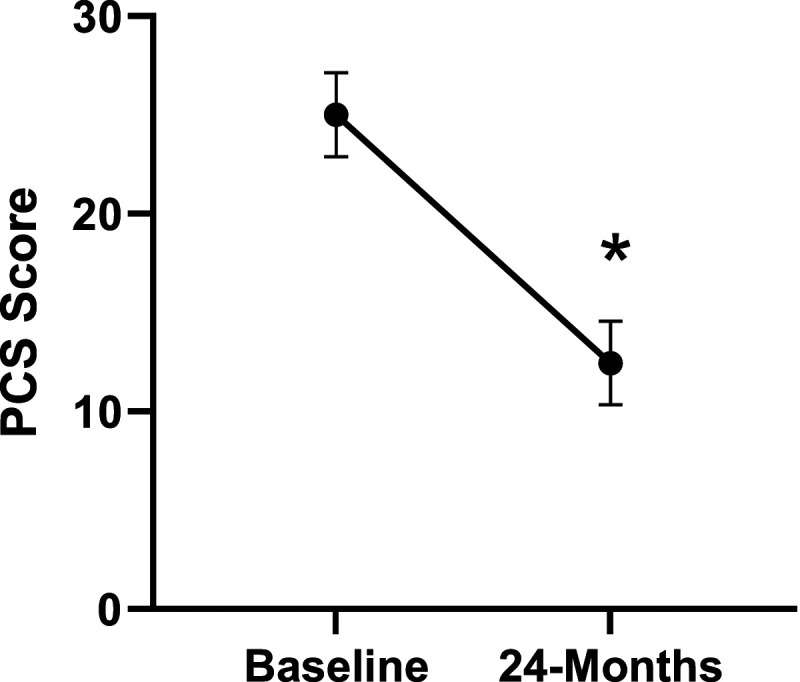
Change in pain catastrophizing from baseline to 24-months follow-up. Error bars represent confidence intervals. *Abbreviations*: PCS, Pain Catastrophizing Scale. *P < .05.

**Figure 6.**
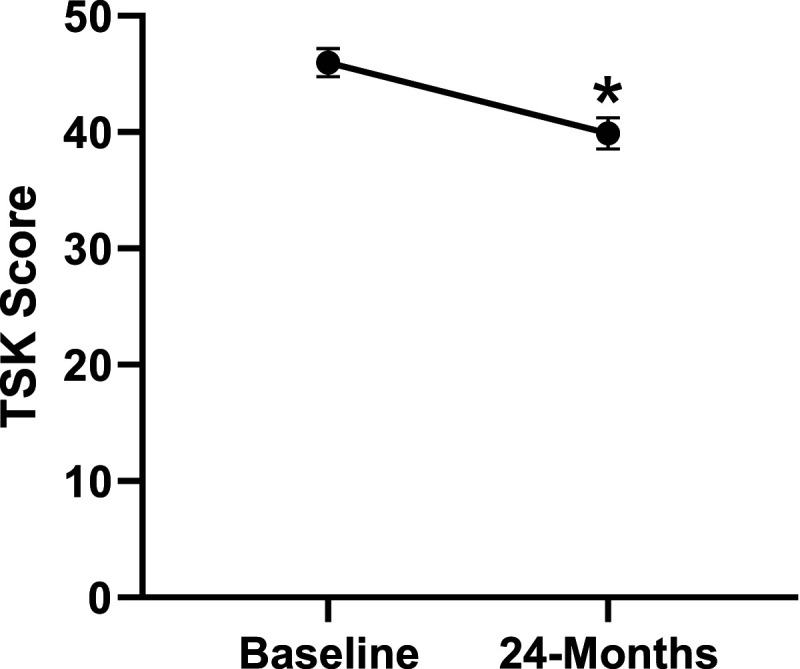
Change in kinesiophobia from baseline to 24-months follow-up. Error bars represent confidence intervals. Abbreviations: TSK, Tampa Scale of Kinesiophobia. *P < .05.

**Figure 7.**
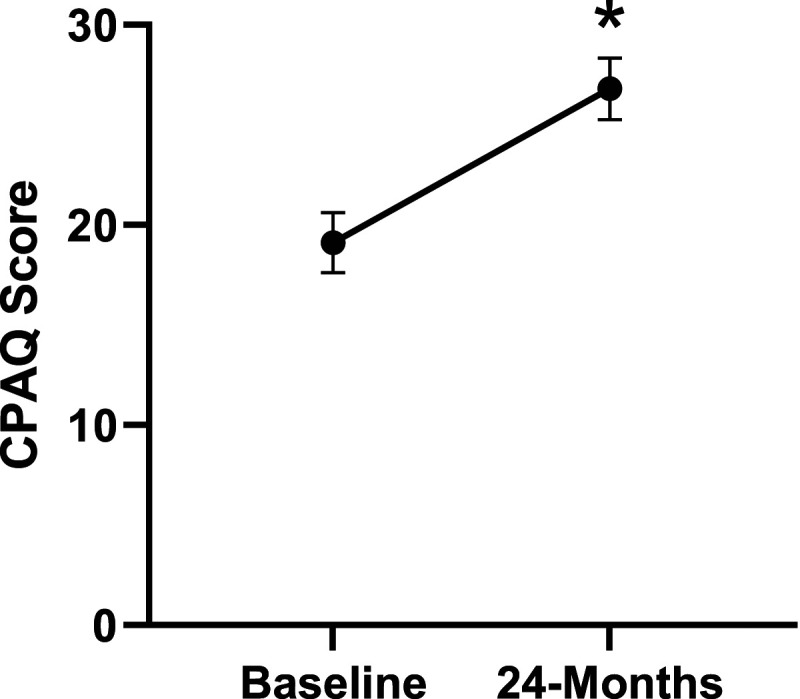
Change in chronic pain acceptance from baseline to 24-months follow-up. Error bars represent confidence intervals. *Abbreviations*: CPAQ, Chronic Pain Acceptance Questionnaire. *P < .05.

**Figure 8.**
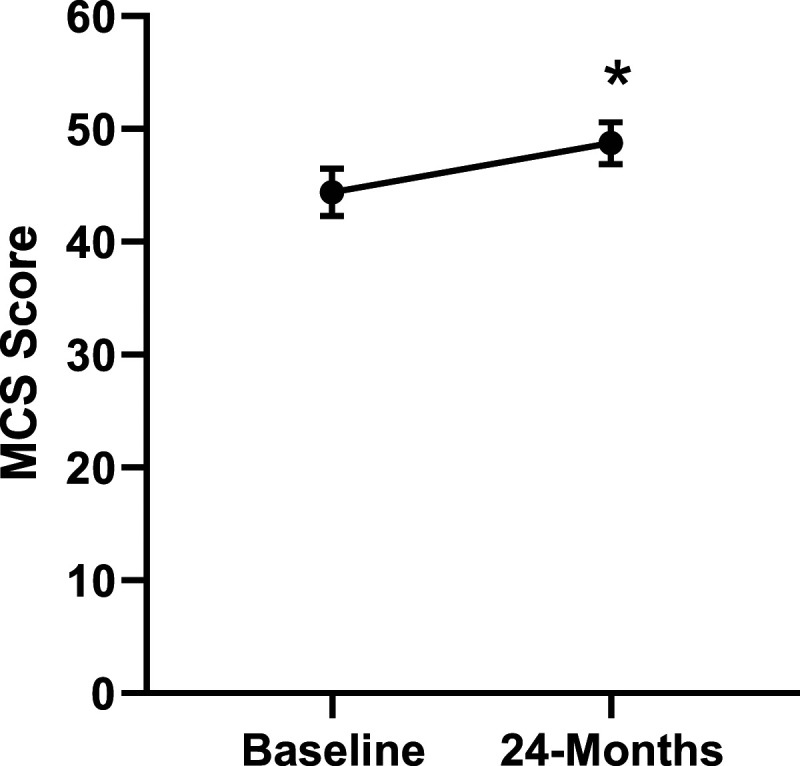
Change in mental health from baseline to 24-months follow-up. Error bars represent confidence intervals. *Abbreviations*: MCS, Mental Component Summary. *P < .05.

**Figure 9.**
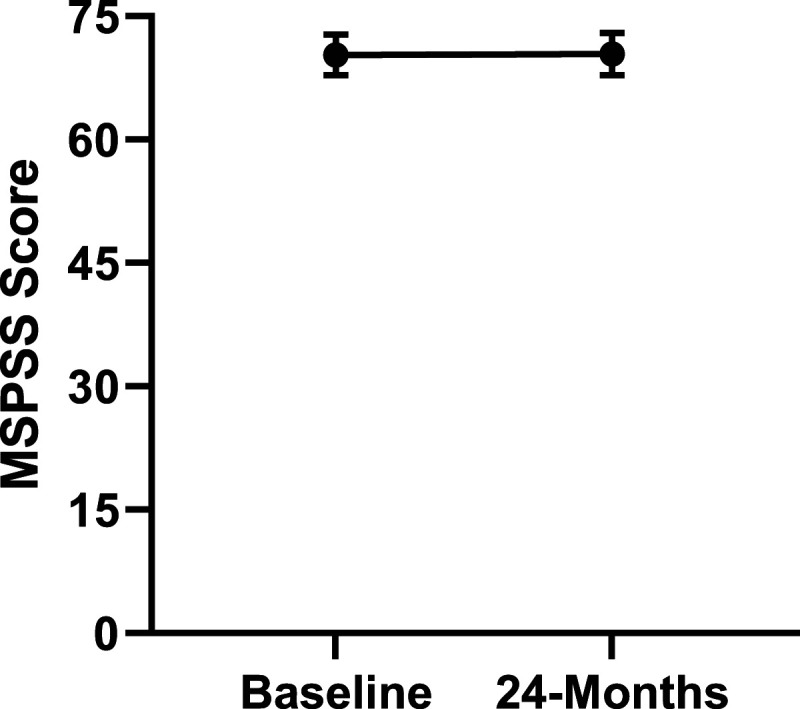
Change in social support from baseline to 24-months follow-up. Error bars represent confidence intervals. *Abbreviations*: MSPSS, Multidimensional Scale of Perceived Social Support. *P* > .05 not significant.

Patient demographics are reported in Table 1. Surgical and intraoperative variables are presented in Table 2. On average, patients who underwent surgery had a significant decrease in scores for NRS-B [*F*(1,128) = 193.67, *P* < .001], NRS-L [*F*(1,128) = 149.75, *P* < .001], and mODI [*F*(1128 = 137.47, *P* < .001] from baseline to 24-months follow-up. Pain Catastrophizing Scale [*F*(1, 128) = 121.61, *P* < .001], CPAQ-8 [*F*(1, 128) = 91.99, *P* < .001], TSK [*F*(1, 128) = 78.94, *P* < .001] and MCS [*F*(1, 128) = 23.381, *P* = .001] scores showed significant improvement. No significant change in MSPSS [*F*(1, 128) = .008, *P* = .930] scores was achieved (see Figures 2-9).

**Table 1.** Baseline Demographic Values.

Variable	N	Percentage (%)
Age (years)	57.8 (SD = 14.6)	
Sex		
Female	92	52
Male	85	48
Education		
Less than high school	35	19.8
High school diploma or greater	135	76.3
Choose not to answer	7	3.9
Smoking status		
Yes	34	19.2
No	143	80.8
Current Work status		
Not employed	28	15.8
Retired	61	34.5
Employed	53	29.9
Employed but not working	24	13.6
Other	11	6.2
Time with the condition		
Under 2 years	66	37.3
Over 2 years	102	57.6
Missing	9	5.1
Total	177	100

**Table 2.** Surgical and Intraoperative Details.

Variable	N	Percentage (%)
Principle pathology		
Disc herniation	47	26.6
Degenerative disk disease	4	2.3
Stenosis	53	29.9
Spondylolisthesis	51	28.8
Deformity	21	11.9
Missing	1	0.6
Chief complaint		
Back pain	10	5.6
Radiculopathy	86	48.6
Myelopathy	1	0.6
Neurogenic claudication	73	41.2
Deformity	6	3.4
Missing	1	0.6
Surgical type		
Discectomy, decompression and fusion	69	39.0
Decompression and fusion	33	18.6
Discectomy and fusion	11	6.2
Discectomy and/or decompression	52	29.4
Fusion alone	6	3.4
Decompression, fusion and osteotomy	2	1.1
Discectomy, decompression, fusion and osteotomy	3	1.7
Fusion and osteotomy	1	0.6
Total	177	100

When looking more closely at patient outcomes, 55.9% of patients reached the meaningful change parameters for mODI compared to 63.3% and 64.4% of patients for NRS-B and NRS-L, respectively.

### Leg Pain

**Figure 10.**
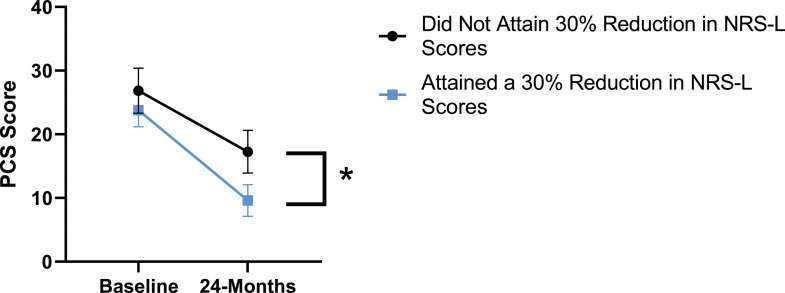
PCS changes categorized by NRS-L outcome, compared by mixed-measures ANOVA. Error bars represent confidence intervals. *Abbreviations*: PCS, Pain Catastrophizing Scale; NRS-L, Numeric Rating Scale for leg pain; ANOVA, analysis of variance. *P < .05.

**Figure 11.**
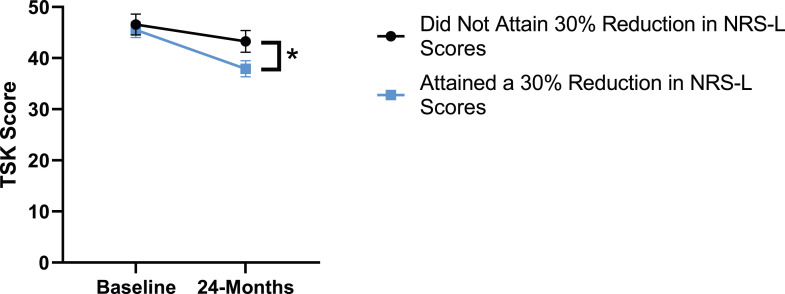
TSK changes categorized by NRS-L outcome, compared by mixed-measures ANOVA. Error bars represent confidence intervals. *Abbreviations*: TSK, Tampa Scale of Kinesiophobia; NRS-L, Numeric Rating Scale for leg pain; ANOVA, analysis of variance. *P < .05.

**Figure 12.**
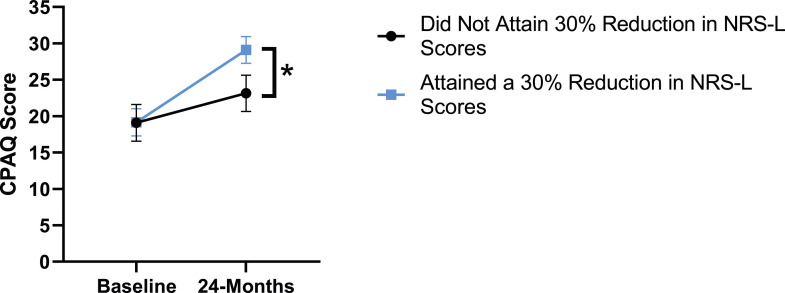
CPAQ changes categorized by NRS-L outcome, compared by mixed-measures ANOVA. Error bars represent confidence intervals. *Abbreviations*: CPAQ, Chronic Pain Acceptance Questionnaire; NRS-L, Numeric Rating Scale for leg pain; ANOVA, analysis of variance. *P < .05.

**Figure 13.**
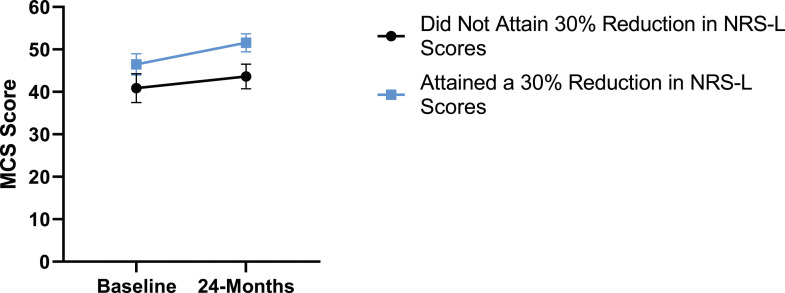
MCS changes categorized by NRS-L outcome, compared by mixed-measures ANOVA. Error bars represent confidence intervals. *Abbreviations*: MCS, Mental Component Summary; NRS-L, Numeric Rating Scale for leg pain; ANOVA, analysis of variance. *P* > .05 not significant.

**Figure 14.**
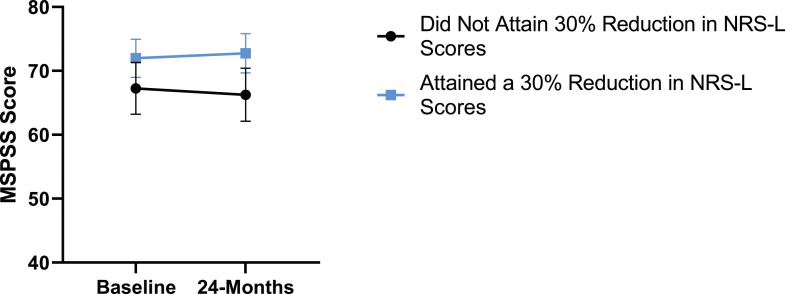
MSPSS changes categorized by NRS-L outcome, compared by mixed-measures ANOVA. Error bars represent confidence intervals. *Abbreviations*: MSPSS, Multidimensional Scale of Perceived Social Support; NRS-L, Numeric Rating Scale for leg pain; ANOVA, analysis of variance. *P* > .05 not significant.

When looking at the 2 surgical cohorts based on NRS-L scores, PCS [*F*(1,129) = 104.32, *P* < .001], TSK [*F*(1,129) = 62.34, *P* < .001], CPAQ-8 [*F*(1,129) = 74.59, *P* < .001] and MCS [*F*(1,129) = 18.05, *P* = .001] were significantly different between baseline and 24-months follow-up while MSPSS [*F*(1,129) = .007, *P* = .934 did not reach significance. The effect of attaining meaningful change for leg pain showed a significant difference for PCS [*F*(1, 129) = 3.89, *P* = .050], TSK [*F*(1, 129) = 9.71, *P* = .01] and CPAQ-8 [*F*(1,129) = 13.30, *P* < .001] while MSPSS [*F*(1, 129) = .498, *P* = .482] and MCS [*F*(1,129) = 1.58, *P* = .212] did not reach significance. This is seen in Figures 10-14.

### Back Pain

**Figure 15.**
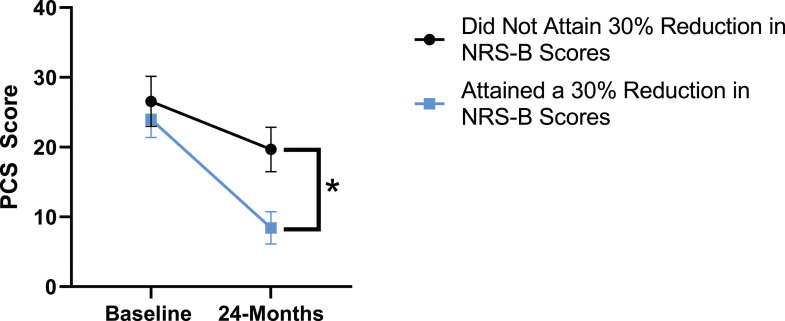
PCS changes categorized by NRS-B outcome, compared by mixed-measures ANOVA. Error bars represent confidence intervals. *Abbreviations*: PCS, Pain Catastrophizing Scale; NRS-B, Numeric Rating Scale for back pain; ANOVA, analysis of variance. *P < .05.

**Figure 16.**
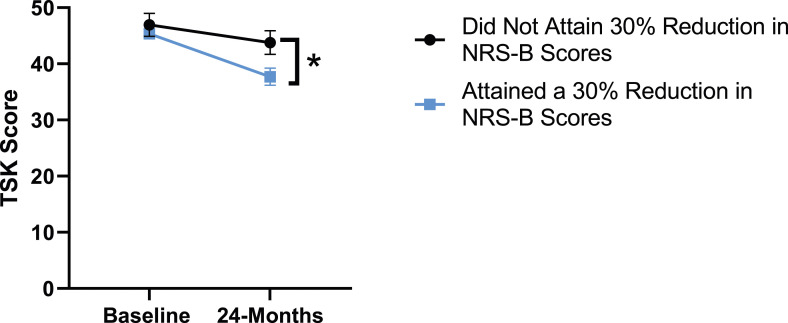
TSK changes categorized by NRS-B outcome, compared by mixed-measures ANOVA. Error bars represent confidence intervals. *Abbreviations*: TSK, Tampa Scale of Kinesiophobia; NRS-B, Numeric Rating Scale for back pain; ANOVA, analysis of variance. *P < .05.

**Figure 17.**
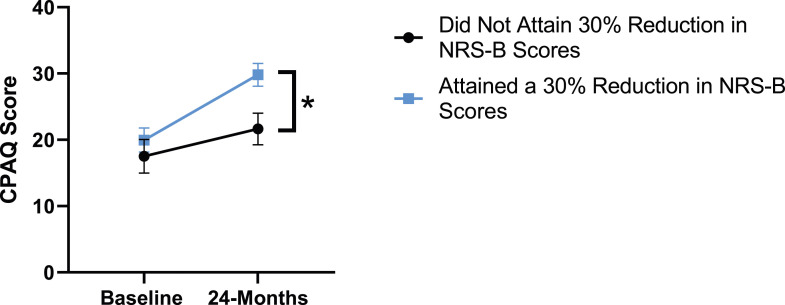
CPAQ-8 changes categorized by NRS-B outcome, compared by mixed-measures ANOVA. Error bars represent confidence intervals. *Abbreviations*: CPAQ, Chronic Pain Acceptance Questionnaire; NRS-B, Numeric Rating Scale for back pain; ANOVA, analysis of variance. *P < .05.

**Figure 18.**
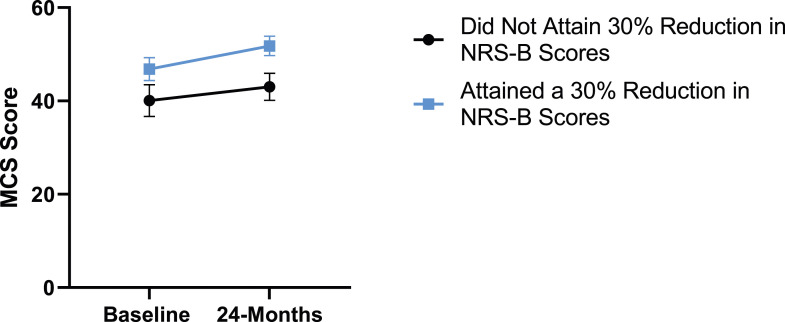
MCS changes categorized by NRS-B outcome, compared by mixed-measures ANOVA. Error bars represent confidence intervals. *Abbreviations*: MCS, Mental Component Summary; NRS-B, Numeric Rating Scale for back pain; ANOVA, analysis of variance. *P* > .05 not significant.

**Figure 19.**
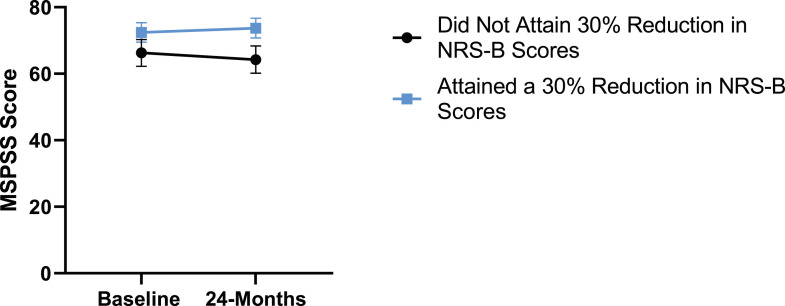
MSPSS changes categorized by NRS-B outcome, compared by mixed-measures ANOVA. Error bars represent confidence intervals. *Abbreviations*: MSPSS, Multidimensional Scale of Perceived Social Support; NRS-B, Numeric Rating Scale for back pain; ANOVA, analysis of variance. *P* > .05 not significant.

When looking at patient outcomes according to scores for back pain, PCS [F (1,129) = 99.50, *P* < .001], TSK [*F*(1,129) = 60.67, *P* < .001], CPAQ-8 [*F*(1,129) = 72.97, *P* < .001] and MCS [*F*(1,129) = 18.09, *P* = .001] improved significantly between timepoints while MSPSS [*F*(1,129) = .08, *P* = .777] did not reach significance. Reaching meaningful change for back pain had a significant effect on PCS [*F*(1,129) = 14.79, *P* < .001], TSK [*F*(1,129) = 10.47, *P* = .01] and CPAQ-8 [*F*(1,129) = 12.16, *P* < .001] while MSPSS [*F*(1,129) = 1.72, *P* = .192] and MCS [*F*(1,129) = 1.17, *P* = .281] did not show significance. This is seen in Figures 15-19.

### Modified Oswestry Disability Index

**Figure 20.**
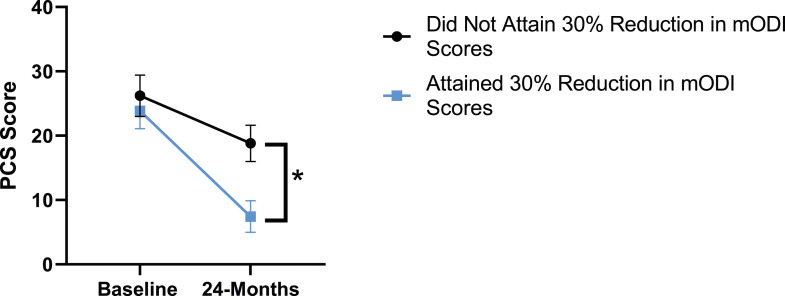
PCS changes categorized by mODI outcome, compared by mixed-measures ANOVA. Error bars represent confidence intervals. *Abbreviations*: PCS, Pain Catastrophizing Scale; mODI, modified Oswestry Disability Index; ANOVA, analysis of variance. *P < .05.

**Figure 21.**
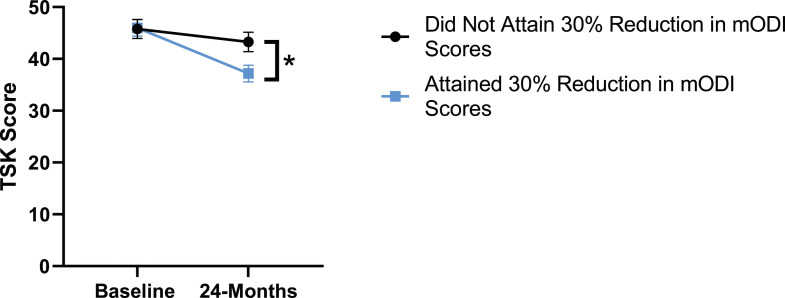
TSK changes categorized by mODI outcome, compared by mixed-measures ANOVA. Error bars represent confidence intervals. *Abbreviations*: TSK, Tampa Scale of Kinesiophobia; mODI, modified Oswestry Disability Index; ANOVA, analysis of variance. *P < .05.

**Figure 22.**
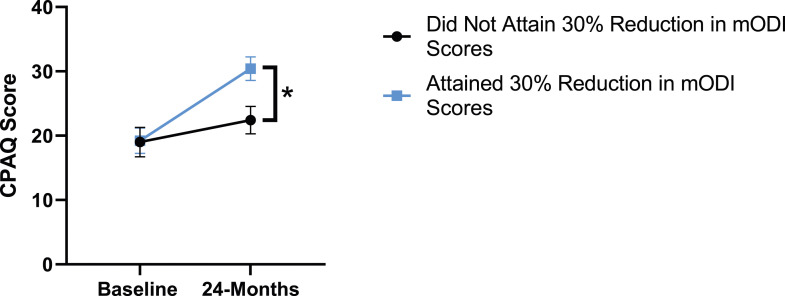
CPAQ-8 changes categorized by mODI outcome, compared by mixed-measures ANOVA. Error bars represent confidence intervals. *Abbreviations*: CPAQ, Chronic Pain Acceptance Questionnaire; mODI, modified Oswestry Disability Index; ANOVA, analysis of variance. *P < .05.

**Figure 23.**
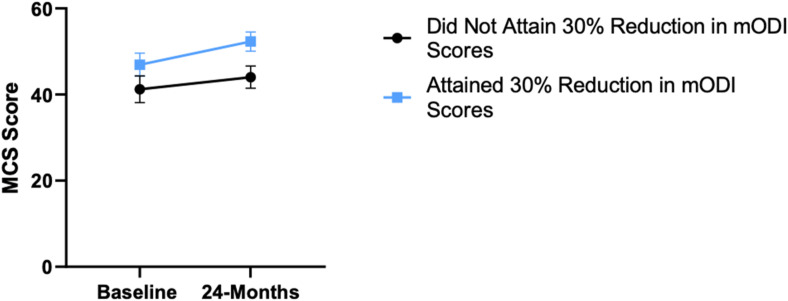
MCS changes categorized by mODI outcome, compared by mixed-measures ANOVA. Error bars represent confidence intervals. *Abbreviations*: MCS, Mental Component Summary; mODI, modified Oswestry Disability Index; ANOVA, analysis of variance. *P* > .05 not significant.

**Figure 24.**
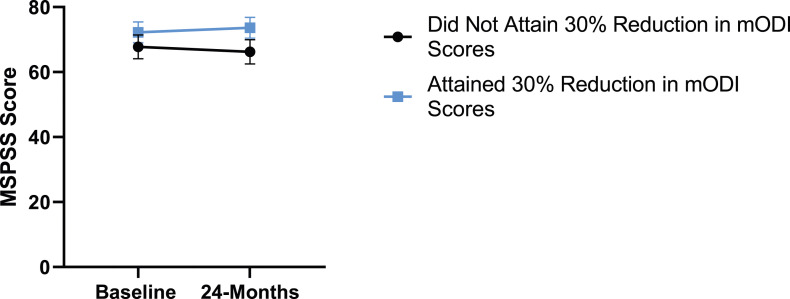
MSPSS changes categorized by mODI outcome, compared by mixed-measures ANOVA. Error bars represent confidence intervals. *Abbreviations*: MSPSS, Multidimensional Scale of Perceived Social Support; mODI, modified Oswestry Disability Index; ANOVA, analysis of variance. *P* > .05 not significant.

Patient outcomes according to mODI scores show a similar trend to the results described for both back and leg pain. Patients had significant improvement in their PCS [*F*(1,129) = 124.49, *P* < .001], TSK [*F*(1,129) = 79.36, *P* < .001], CPAQ-8 [*F*(1,129) = 96.12, *P* < .001) and MCS [*F*(11 129) = 21.09, *P* = .001] between timepoints while MSPSS [*F*[1129) = .002, *P* = .967] did not reach significance. The effect of attaining meaningful change for mODI on PCS [*F*(1,129) = 17.91, *P* < .001], TSK [*F*(1,129) = 24.89, *P* < .001], CPAQ-8 [*F*(1,129) = 27.36, *P* < .001], while MSPSS [*F*(1,129) = 1.42, *P* = .236] and MCS [*F*(1,129) = 2.04, *P* = .156] were not significant. This is demonstrated in Figures 20-24.

### Patient Expectations

Of the spine surgery patient population under investigation, 94.1% expected their mental health to improve after surgery. At 24-months follow-up, 75.88% of all patients reported that the surgery met their expectations for mental health improvement.

**Figure 25.**
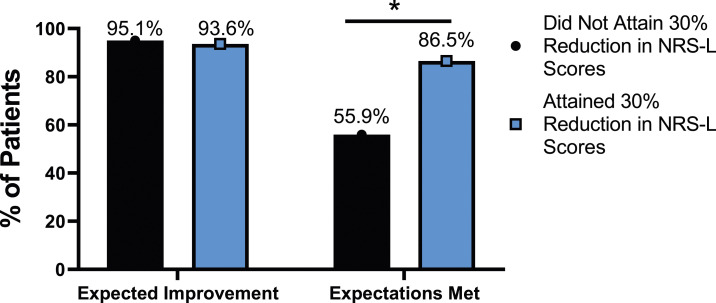
Expectations for mental health improvement categorized by NRS-L outcome, compared by chi-square test. *Abbreviations*: NRS-L, Numeric Rating Scale for leg pain. *P < .05.

**Figure 26.**
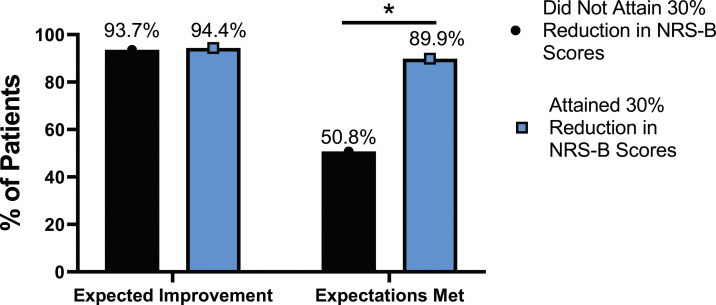
Expectations for mental health improvement by NRS-B outcome, compared by chi-square test. *Abbreviations*: NRS-B, Numeric Rating Scale for back pain. *P < .05.

**Figure 27.**
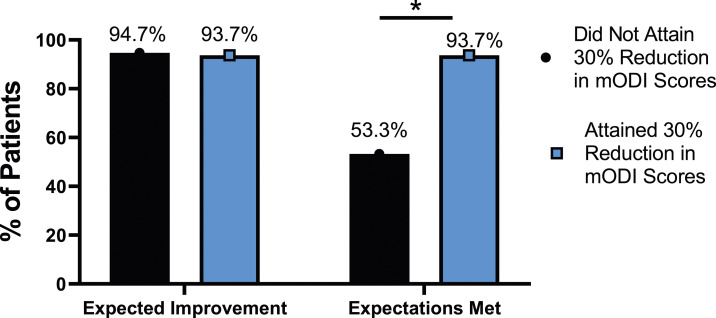
Expectations for mental health improvement by mODI outcome, compared by chi-square test. *Abbreviations*: mODI, modified Oswestry Disability Index. *P < .05.

The rate of expectation for improvement in mental health was similar, regardless of meaningful change achievement for mODI [*X*^2^ (4, N = 170) = 2.02, *P* = .731], NRS back pain [*X*^2^ (4, N = 170) = 3.64, *P* = .457], or NRS leg pain [*X*^2^ (4, N = 170) = 5.63, *P* = .229]. However, at 24-months follow-up, patients who did not meet the meaningful change parameters for mODI [*X*^2^ (4, N = 170) = 56.31, *P* < .001], NRS back pain [*X*^2^ (4, N = 170) = 49.77, *P* < .001] or NRS leg pain [*X*^2^ (4, N = 170) = 30.95, *P* < .001] were significantly less likely to report that their expectations were met for improvement in mental health. This is seen in Figures 25-27.

## Discussion

The current results show that, on average, patients undergoing spine surgery show significant improvement on back and leg pain, disability, pain catastrophizing, kinesiophobia and chronic pain acceptance scales. However, this study goes further than looking at average outcomes and compares the effect of meaningful change attainment in disability and both leg and back pain on various psychometric scores for patients undergoing thoracolumbar surgery. On average, 55.9% - 64.4% of patients attained meaningful change for pain or disability. This is consistent with a national CSORN trajectory study which reported that 58.4%-70.7% of patients who underwent surgery for lumbar spine stenosis had a good or excellent outcome.^10^

Our study shows that social support did not change significantly between baseline and follow-up, regardless of attainment of meaningful change. This is consistent with previous literature which has found that social support does not change significantly postoperatively.^23^ The interpretation for the MCS findings is less clear. The current study showed that MCS changed significantly between baseline and 24-months follow-up but was not significant based on attainment of meaningful change. This is in keeping with previous literature that has been conflicted on MCS and operative outcomes. One study found that a lower preoperative MCS predicts a greater improvement in ODI and PCS postoperatively^24^ while another did not find MCS to predict patient-reported outcomes.^25^ It has been established that general mental health is impacted by a multitude of variables including comorbidities, psychiatric conditions, active compensation case and smoking status.^25,26^

Rahman et al (2020) also found that all patients, regardless of if they had improving, worsening or new onset anxiety/depression saw some improvements in their patient-reported outcome measurement information system scores, however the degree of improvement varied depending on severity of psychological worsening.^27^ Further, Bekeris et al (2020) found that after spine surgery, 6% and 11% of patients developed a new onset depression or anxiety, respectively,^28^ indicating that a proportion of patients had psychological worsening. We did not find worsening of mean psychometric scores, regardless of surgical outcome. However, we relied on self-reported psychometric scores instead of psychological diagnoses, which could explain these differences. Our study adds to the existing literature which demonstrates the complex relationships between surgical outcomes and psychological health. Patients who achieved a 30% reduction in their disability or pain scores saw significant improvement in their psychometric scores when compared to those who did not achieve this 30% benchmark. It is reassuring that patients who did not achieve meaningful change, on average, did not report worse psychometric scores at 24-months. Future research could benefit from focusing on more specific clinical variables as our study did not look at psychological diagnoses.

Most patients (94.1%) expected their spine surgery to improve their mental health, however, patients who did not achieve a 30% reduction in their pain or disability were significantly less likely to report having their expectations met for mental health improvement. This is important because research has shown that patients who had their general surgical expectations met were more likely to also report improvements in functional status.^7^ A previous study found that 81% of patients had their general surgical expectations fulfilled,^7^ which is similar to the rate of mental health surgical expectation fulfillment in the group who achieved minimal change (86.5%-93.7%), but not those who did not achieve this benchmark. Interestingly, in Yee et al’s (2008) study, patients who did not have their general surgical expectations met reported less improvement in their ODI scores.^7^ Although Yee et al looked at this relationship in reverse, their results support our findings that when patients do not have their surgical expectations met, they also lack improvements in their ODI scores.

The limitations in this study include using a broad study population containing different types of spinal pathologies and surgical approaches. This was beneficial to study the surgical population as a whole, however, certain pathologies may have unique impacts on mental well-being. Stratification based on pathology or surgical type may be of interest in future studies. Further, we did not look at specific psychological diagnoses such as anxiety and depression, which may provide additional insight to the complex relationship between spine surgery outcomes and psychological health.

## Conclusion

On average, patients see a significant improvement in their leg and back pain, disability, pain catastrophizing, kinesiophobia and chronic pain acceptance following thoracolumbar spine surgery. On average, patients who do not meet parameters for meaningful change do not show worsening of the psychometric scores measured in this study. The majority of patients expect surgery to improve their mental health and patients are significantly more likely to report fulfillment of their expectations if they attain meaningful change in their pain/disability scores.

## Data Availability

The datasets used and/or analyzed during the current study are available from the corresponding author on reasonable request.

## References

[1] Tripp D Abraham E Lambert M , et al. Biopsychosocial factors predict quality of life in thoracolumbar spine surgery. Qual Life Res. 2017;26(11):3099-3110. 28730301 10.1007/s11136-017-1654-x

[2] Flanigan DC Everhart JS Glassman AH . Psychological factors affecting rehabilitation and outcomes following elective orthopaedic surgery. J Am Acad Orthop Surg. 2015;23(9):563-570. doi:10.5435/JAAOS-D-14-00225 26195567

[3] Coronado RA George SZ Devin CJ Wegener ST Archer KR . Pain sensitivity and pain catastrophizing are associated with persistent pain and disability after lumbar spine surgery. Arch Phys Med Rehabil. 2015;96(10):1763-1770. doi:10.1016/j.apmr.2015.06.003 26101845 PMC4601931

[4] Havakeshian S Mannion AF . Negative beliefs and psychological disturbance in spine surgery patients: A cause or consequence of a poor treatment outcome? Eur Spine J. 2013;22(12):2827-2835. doi:10.1007/s00586-013-2822-5 23695229 PMC3843780

[5] Rosenberger PH Jokl P Ickovics J . Psychosocial factors and surgical outcomes: An evidence-based literature review. J Am Acad Orthop Surg. 2006;14(7):397-405. doi:10.5435/00124635-200607000-00002 16822887

[6] McCracken LM Eccleston C . Coping or acceptance: What to do about chronic pain? Pain. 2003;105(1-2):197-204. doi:10.1016/s0304-3959(03)00202-1 14499436

[7] Yee A Adjei N Do J Ford M Finkelstein J . Do patient expectations of spinal surgery relate to functional outcome? Clin Orthop Relat Res. 2008;466(5):1154-1161. doi:10.1007/s11999-008-0194-7 18347892 PMC2311462

[8] Ferreira-Valente MA Pais-Ribeiro JL Jensen MP . Validity of four pain intensity rating scales. Pain. 2011;152(10):2399-2404. doi:10.1016/j.pain.2011.07.005 21856077

[9] Karcioglu O Topacoglu H Dikme O Dikme O . A systematic review of the pain scales in adults: Which to use? Am J Emerg Med. 2018;36(4):707-714. doi:10.1016/j.ajem.2018.01.008 29321111

[10] Hebert JJ Abraham E Wedderkopp N , et al. Patients undergoing surgery for lumbar spinal stenosis experience unique courses of pain and disability: A group-based trajectory analysis. PLoS One. 2019;14(11):e0224200. doi:10.1371/journal.pone.0224200 31697714 PMC6837529

[11] Fairbank JC Pynsent PB . The oswestry disability index. Spine (Phila Pa 1976). 2000;25(22):2940-2952. doi:10.1097/00007632-200011150-00017 11074683

[12] Vianin M . Psychometric properties and clinical usefulness of the Oswestry disability index. J Chiropr Med. 2008;7(4):161-163. doi:10.1016/j.jcm.2008.07.001 19646379 PMC2697602

[13] Cheak-Zamora NC Wyrwich KW McBride TD . Reliability and validity of the SF-12v2 in the medical expenditure panel survey. Qual Life Res. 2009;18(6):727-735. doi:10.1007/s11136-009-9483-1 19424821

[14] Ware J Jr Kosinski M Keller SD . A 12-item short-form health survey: Construction of scales and preliminary tests of reliability and validity. Med Care. 1996;34(3):220-233. doi:10.1097/00005650-199603000-00003 8628042

[15] Sullivan MJL Bishop SR Pivik J . The pain catastrophizing scale: Development and validation. Psychol Assess. 1995;7(4):524-532. doi:10.1037/1040-3590.7.4.524

[16] Miller RP Kori SH Todd DD . The Tampa scale: A measure of kinisophobia. Clin J Pain. 1991;7(1):51.

[17] Vlaeyen JWS Kole-Snijders AMJ Boeren RGB van Eek H . Fear of movement/(re)injury in chronic low back pain and its relation to behavioral performance. Pain. 1995;62(3):363-372. doi:10.1016/0304-3959(94)00279-N 8657437

[18] Baranoff J Hanrahan SJ Kapur D Connor JP . Validation of the chronic pain acceptance questionnaire-8 in an Australian pain clinic sample. Int J Behav Med. 2014;21(1):177-185. doi:10.1007/s12529-012-9278-6 23179676

[19] Fish RA McGuire B Hogan M Morrison TG Stewart I . Validation of the chronic pain acceptance questionnaire (CPAQ) in an Internet sample and development and preliminary validation of the CPAQ-8. Pain. 2010;149(3):435-443. doi:10.1016/j.pain.2009.12.016 20188472

[20] Zimet GD Dahlem NW Zimet SG Farley GK . The multidimensional scale of perceived social support. J Pers Assess. 1988;52(1):30-41. 10.1080/00223891.1990.9674095 2280326

[21] Zimet GD Powell SS Farley GK Werkman S Berkoff KA . Psychometric characteristics of the multidimensional scale of perceived social support. J Pers Assess. 1990;55(3-4):610-617. doi:10.1080/00223891.1990.9674095 2280326

[22] Asher AM Oleisky ER Pennings JS , et al. Measuring clinically relevant improvement after lumbar spine surgery: Is it time for something new? Spine J. 2020;20(6):847-856. doi:10.1016/j.spinee.2020.01.010 32001385

[23] Adogwa O Elsamadicy AA Vuong VD , et al. Effect of social support and marital status on perceived surgical effectiveness and 30-day hospital readmission. Global Spine J. 2017;7(8):774-779. doi:10.1177/2192568217696696 29238642 PMC5721993

[24] Carreon LY Glassman SD Djurasovic M , et al. Are preoperative health-related quality of life scores predictive of clinical outcomes after lumbar fusion? Spine (Phila Pa 1976). 2009;34(7):725-730. doi:10.1097/BRS.0b013e318198cae4 19333106

[25] Asher R Mason AE Weiner J Fessler RG . The relationship between preoperative general mental health and postoperative quality of life in minimally invasive lumbar spine surgery. Neurosurgery. 2015;76(6):672-679. doi:10.1227/NEU.0000000000000695 25714515

[26] Slover J Abdu WA Hanscom B Weinstein JN . The impact of comorbidities on the change in short-form 36 and oswestry scores following lumbar spine surgery. Spine (Phila Pa 1976). 2006;31(17):1974-1980. doi:10.1097/01.brs.0000229252.30903.b9 16924216

[27] Rahman R Ibaseta A Reidler JS , et al. Changes in patients' depression and anxiety associated with changes in patient-reported outcomes after spine surgery. J Neurosurg Spine. 2020:1-20 [published online ahead of print, 2020 Jan 31]. doi:10.3171/2019.11.SPINE19586 32005017

[28] Bekeris J Wilson LA Fiasconaro M , et al. New onset depression and anxiety after spinal fusion surgery: Incidence and risk factors. Spine (Phila Pa 1976). 2020;45(16):1161-1169. doi:10.1097/BRS.0000000000003467 32150130

